# Biomimetic Cell Membrane-coated Nanovaccines in Anti-tumor Immunotherapy

**DOI:** 10.7150/thno.128136

**Published:** 2026-03-03

**Authors:** Yizhang Chen, Weiyue Zhang, Xin Huang

**Affiliations:** 1 Department of Orthopaedics, Union Hospital, Tongji Medical College, Huazhong University of Science and Technology, Wuhan 430022, China.; 2 Department of Endocrinology, Union Hospital, Tongji Medical College, Huazhong University of Science and Technology, Wuhan 430022, China.; 3 Hubei Key Laboratory of Regenerative Medicine and Multi-disciplinary Translational Research (Huazhong University of Science and Technology), Wuhan, Hubei 430022, China.

**Keywords:** cell membrane coating, biomimetic nanoparticles, nanomedicine, tumor immunotherapy, cancer vaccine.

## Abstract

Biomimetic nanovaccines have recently emerged as a frontier in vaccine development. These nanovaccines are designed to structurally and morphologically mimic natural pathogens, including bacteria, viruses, and certain eukaryotic cells. Engineered to replicate pathogenic surface characteristics, these nanoplatforms improve targeted delivery to antigen-presenting cells (APCs) and prolong systemic circulation, which in turn enhances antigen presentation and promotes stronger adaptive immune activation. The preparation process for biomimetic nanovaccines is also highlighted, involving isolating and purifying source cell membrane, encapsulation of synthetic nanoparticle core, and verifying. Additionally, this study offers a thorough evaluation of various biomimetic nanovaccines, particularly those with cell membrane coatings derived from tumor cells, immune cells, or bacteria. Furthermore, we also assess their capabilities when combined with common treatment modalities, including immune checkpoint inhibitors, chemotherapy, and photothermal therapy, to achieve anti-tumor effects. The study also discusses the path to clinical translation and existing challenges in manufacturing, safety, and regulatory approval.

## Introduction

Tumor vaccines, which have advanced rapidly in recent years, offer several distinct advantages. First, it offers abundant and specific tumor antigens to activate immune system which could largely avoid immune evasion and heterogeneity 1. It could also activate immunological memory for long term protection. Another advantage is flexibility. Using bioinformatics methods such as antigenic epitope prediction and mutational load to select antigens with higher immunogenicity which could maximize the efficiency 2. Additionally, tumor vaccine could integrate with other anti-tumor therapy. It could not only act as carriers to deliver chemotherapeutic drugs 3, but also change tumor status for better immune checkpoint therapy outcome 4. Currently, a variety of tumor vaccine platforms have been developed (Table 1).

Despite clinical successes, tumor vaccines still face significant challenges. Their limited therapeutic efficacy is largely due to pronounced tumor heterogeneity and a complex tumor microenvironment 12. Besides, accumulating evidence indicates that the inadequate transport of tumor vaccines to lymphoid organs is a key factor hindering the prompt initiation of anti-tumor immunity, which limit its use and efficiency 13. Therefore, enhancing the precision of delivery to tumor sites is essential to mitigate associated systemic toxicity 14.

*In situ* vaccination (ISV) and personalized neoantigen-based vaccines are two notable strategies that have been developed to enhance vaccine efficacy 15, 16. The idea of ISV is to use a specific method, such as drugs, viruses, or radiation, to kill tumor cells and release specific tumor antigens locally, thereby systemic triggering immune response and remodeling TME 17. The key difference between ISV and traditional vaccines lies in their mechanism of immune stimulation. ISV initiates a response by directly inducing ICD in tumor cells 18, while traditional vaccines use mimicked or specific antigens to activate antigen presenting cells (APCs). A distinguished advantage of ISV is its ability to bypass antigen identification and isolation, thereby avoiding the delays and high costs of exogenous personalized vaccine production 19. Personalized neoantigen-based vaccines represent a promising frontier in cancer immunotherapy. Unlike traditional therapeutic vaccines that primarily target widely expressed tumor-associated antigens (TAAs), these personalized approaches are designed against novel epitopes generated by somatic mutations. This approach can effectively circumvent both central and peripheral tolerance mechanisms against self-antigens, thereby reducing the risk of severe autoimmune toxicity 20. However, given that both approaches demand a precise targeted delivery, biomimetic nanovaccines have become a platform for advancing these immunotherapeutic approaches. 21, 22.

To fully maximize delivery efficiency, researchers are increasingly turning to the nanoparticles (NPs). Nanomaterials guarantee a strong delivery capability due to its unique size, which enables lymph nodes to better uptake the vaccine 23. The co-delivery of high heterogeneity antigens and adjuvants is another advantage, which is essential in prompting an immune response 24. Nanocarriers could be engineered to respond to specific TME stimuli, ensuring controlled drug release at the target site 25. However, in lymph nodes, most nanoparticles struggle to penetrate the T-cell-rich paracortex effectively, severely limiting the effective activation of cellular immunity 26. Furthermore, conventional nanovaccines depend on adjuvants to enhance immunogenicity and often elicit a weak cell-mediated immune response, thereby requiring multiple doses.

Several reviews have summarized distinct types of tumor vaccines. For instance, membrane-coated nanovaccines are widely employed as targeted carriers for chemotherapeutic drugs and neoantigens 27; STING-based nanovaccines demonstrate the capacity to reverse the immunosuppressive tumor microenvironment 28; and DCs-targeted nanovaccines enable precise dendritic cell activation 29. Recent studies have increasingly sought inspiration from natural systems to enhance the functionality and efficacy of nanoplatforms 30. The encapsulation of vaccines within cell-derived membranes enhances NPs targeting specificity towards lymph nodes and tumor cells 31. Furthermore, the customizable nature of these membranes facilitates the design of personalized vaccine platforms and combination with other tumor therapy. Theoretically, biomimetic membrane coating is a technology that combines the targeted delivery capacity of membrane coating with the accurate activation of DCs-targeted vaccines.

**Figure 1:**
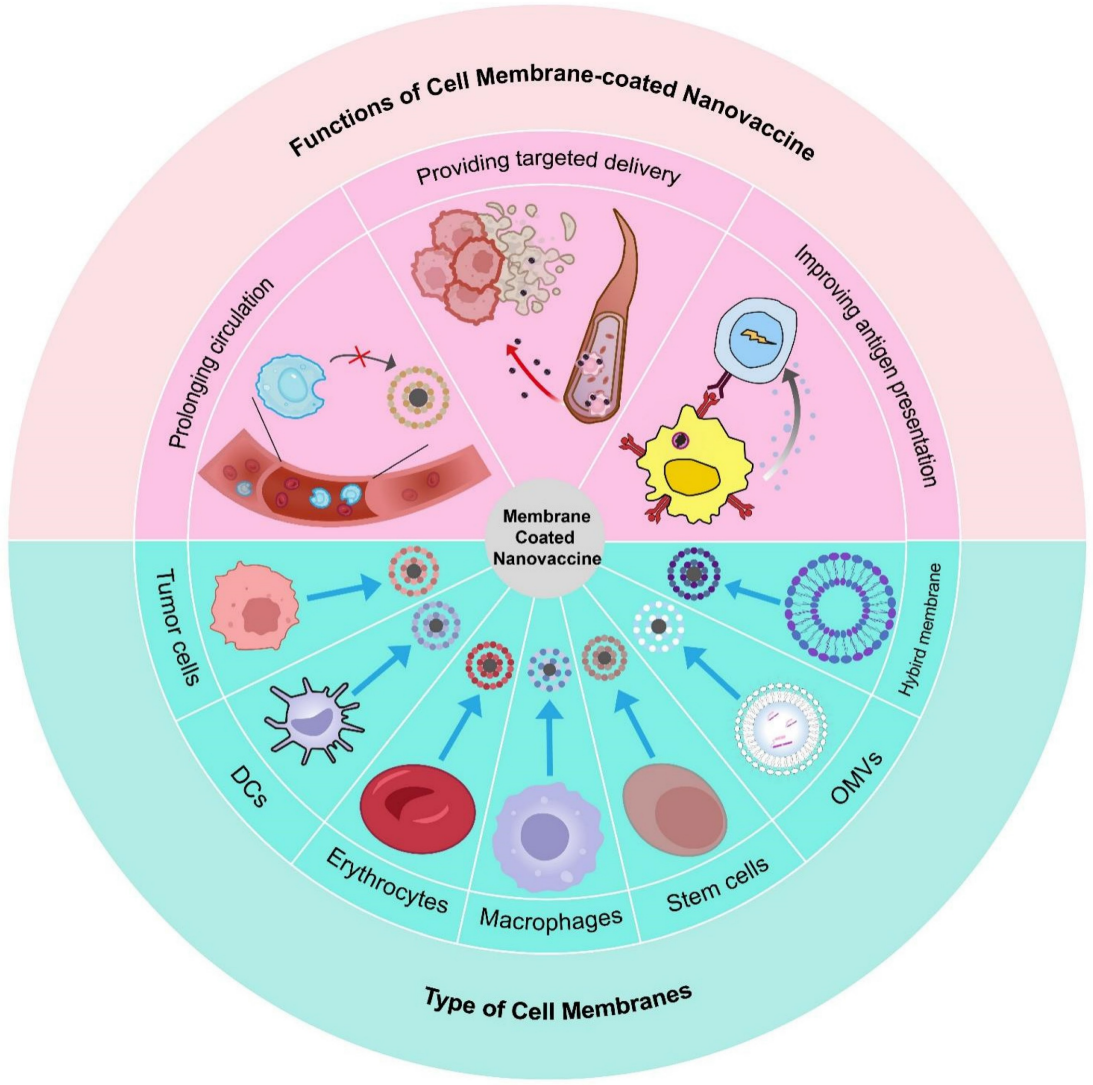
** Schematic of types and sources of biomimetic nanovaccines and their features**. In this review, we discuss some of the most commonly utilized cell membrane in NPs application. Membranes of the NPs derived from different types of cells envelop diverse core to enhance its efficacy. Compared with conventional vaccine approaches, this strategy offers a better targeted delivery, a prolonged circulation, and specific antigen presentation.

In this review, we discuss the fundamental categories of biomimetic cell membrane-coated nanovaccines and their applications in tumor immunotherapy (Figure 1). Additionally, we also discuss the combination with common antitumor strategies, along with present obstacles that may affect future clinical transformation. In challenges and future perspective, we provide a detailed discussion of Good Manufacturing Practice (GMP) in biomimetic cell membrane-coated nanovaccine, a topic that has been less commonly addressed in earlier reviews.

## Nanovaccines in tumors

**Figure 2:**
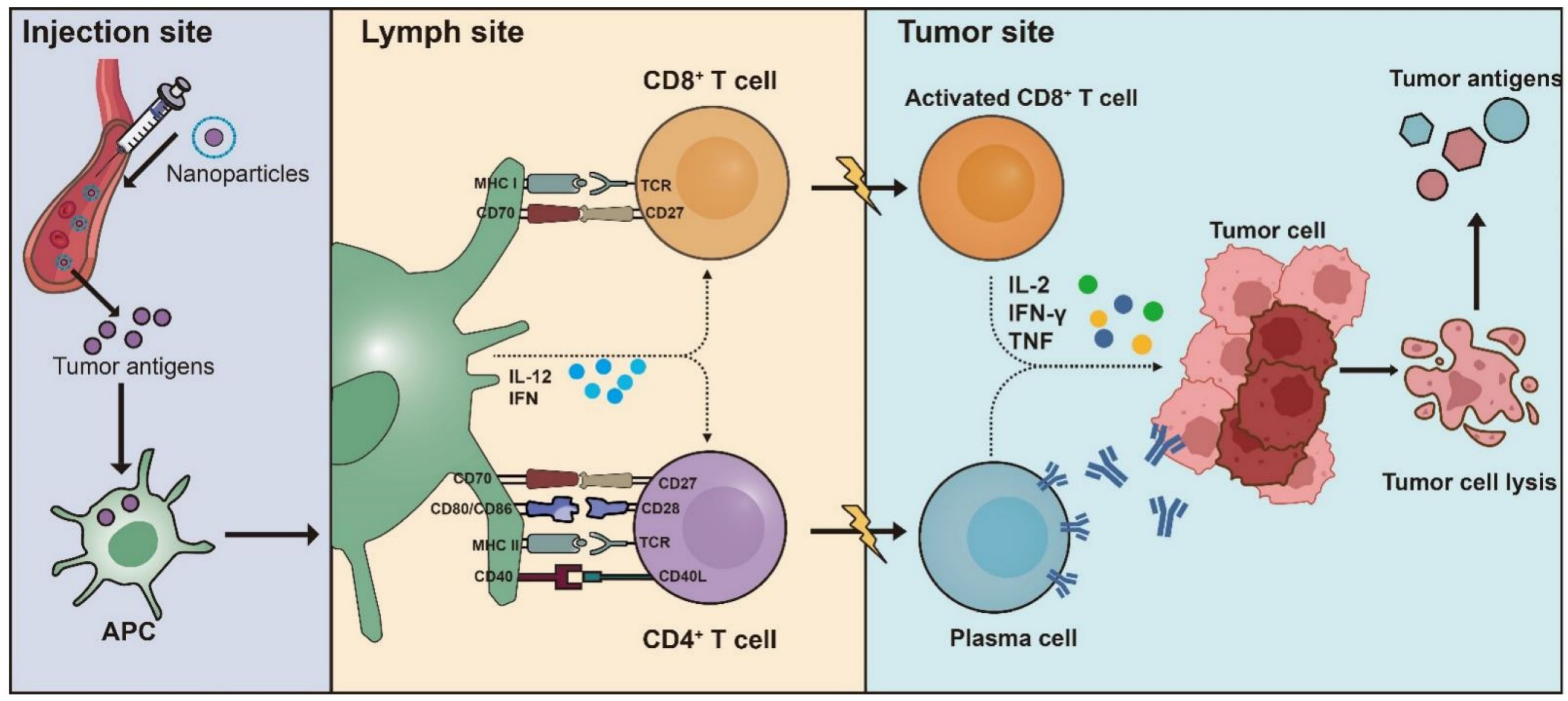
** Basic mechanisms of vaccine.** The process of tumor antigen presentation begins with the interaction of APCs. Following initial antigen capture, the antigen-loaded APCs migrate through the lymphatic system to the draining lymph nodes, which serve as the primary location for T cell priming. In lymph nodes, mature DCs present tumor-derived peptides on major histocompatibility complex (MHC) class I and class II molecules to CD8^+^ and CD4^+^ T cells respectively, including naive T cell populations. A secondary costimulatory signal is essential for full T cell activation, which can be transduced through several distinct receptor-ligand interactions. Key pathways facilitating this signal include CD28 on T cells with CD80 or CD86 on antigen-presenting cells, as well as the interactions between CD27 and CD70, and between CD40 ligand (CD40L) and CD40. These interactions promote tumor-specific T-cell responses, which is further boosted by DCs secretion of interleukin-12 (IL-12) and type I interferons (IFNs). Collectively, these interactions foster the generation and proliferation of activated tumor-specific CD4^+^ and CD8^+^ T cell populations. These T cells migrate to the tumor site, where they recognize their specific antigens and exert cytotoxic effects on tumor cells, producing effector cytokines such as IFN-γ and tumor necrosis factor (TNF). The destruction of tumor cells results in the release of additional tumor antigens, which can be captured, processed, and presented by APCs, thereby inducing polyclonal T cell responses.

The aim of therapeutic tumor vaccines is to elicit a targeted adaptive immune response against tumor-specific antigens, thereby reestablishing control over tumor proliferation, promoting the regression of existing tumors, and eliminating minimal residual disease 32. The most important part of a tumor vaccine is its antigen, which determines the therapeutic effect of the vaccine. Antigens are primarily sourced in two ways: traditional purified antigens and an *in situ* approach that derives them from the tumor itself 33. Another essential component is the vaccine formulation, which includes adjuvants and delivery vesicles (Figure 2) 34. The vaccine formulation platform is designed to deliver or generate antigens to activate T-cell immunity. It encompasses various types, such as protein-based, nucleic acid-based, and vector-based, each selected based on therapeutic intent and tumor characteristics. To improve the therapeutic effect, adjuvants are added into tumor vaccine to enhance antigen presentation and cell maturation. Besides, delivery vehicles could protect the antigen from being cleared in the body and improve its ability to targeting specific tissue 35.

Nanomaterials have been introduced to activate immune system due to limited targeting capability of current tumor immunotherapy drugs and is ineffective against immune tolerance 36. An optimal tumor vaccine is designed to induce a robust, specific, and durable adaptive immune response, which is essential for effectively targeting the heterogeneity and complexity of malignant tumors 37. After administering a nanovaccine, a critical sequence of events called the LDIMP cascade takes place: (L) Loading of tumor-specific antigens onto a nanoscale delivery system; (D) Drainage to and accumulation within lymph nodes; (I) Internalization by resident DCs; (M) Maturation of DCs activated by co-stimulatory signals; and (P) Presentation of tumor antigen-MHC complexes to naive T cells, thereby initiating a adaptive immune response 38. With its unique size, NPs could improve circulation time and provide targeted delivery 39. Additionally, surface charge plays a critical role in modulating the interactions between NPs vaccines and components in the TME 40. Positively charged NPs exhibit enhanced cellular uptake due to electrostatic interactions with the anionic constituents of cell membranes. While this character promotes internalization and targeted delivery, it may also accelerate clearance from blood. In contrast, neutrally or moderately negatively charged NPs exhibit a prolonged circulation time due to reduced interactions with serum proteins, which consequently enhanced their passive accumulation at target sites 41. Additionally, NPs show enhanced an permeability and retention (EPR) effect, leading to their accumulation at the tumor site 42. Research indicates that NPs could activate adverse immune responses and function as intrinsic adjuvants, potentially reducing the require for additional adjuvant doses 43.

Membrane-coated nanovaccines represent a major therapeutic strategy with broad application potential 44. The cellular membrane functions as both a protective barrier and a critical mediator in numerous biological processes, including cell adhesion, signal transduction, and immunological recognition 45. Enveloped NPs with biomimetic cell membrane, such as tumor cell membrane and hybrid membrane, could significantly improve biocompatibility 46. This enhanced biocompatibility allows the vaccine to circulate for a prolonged duration, leading to lower rates of clearance and higher utilization efficiency 47. Additionally, mimicking tumor cells or natural cells allows better recognition between vaccine and APCs which offers high efficiency in boosting T cells. NPs could be modified to extend function 48. For example, strategically designing NPs to target and disrupt key components of the immunosuppressive TME offers a promising approach for TME remodeling and improving immunotherapy efficacy 49. Traditional membrane-coated nanovaccines exhibit strong targeting ability while suffer from poor delivery efficiency. Modifying membrane with structure with chemokine receptor could enhance NPs delivering ability 50. Mannose and adjuvants could also be applied to specific bind receptors on APCs to enhance stimulation effect 51.

## The preparation processes of cell membrane-coated nanovaccines

**Figure 3:**
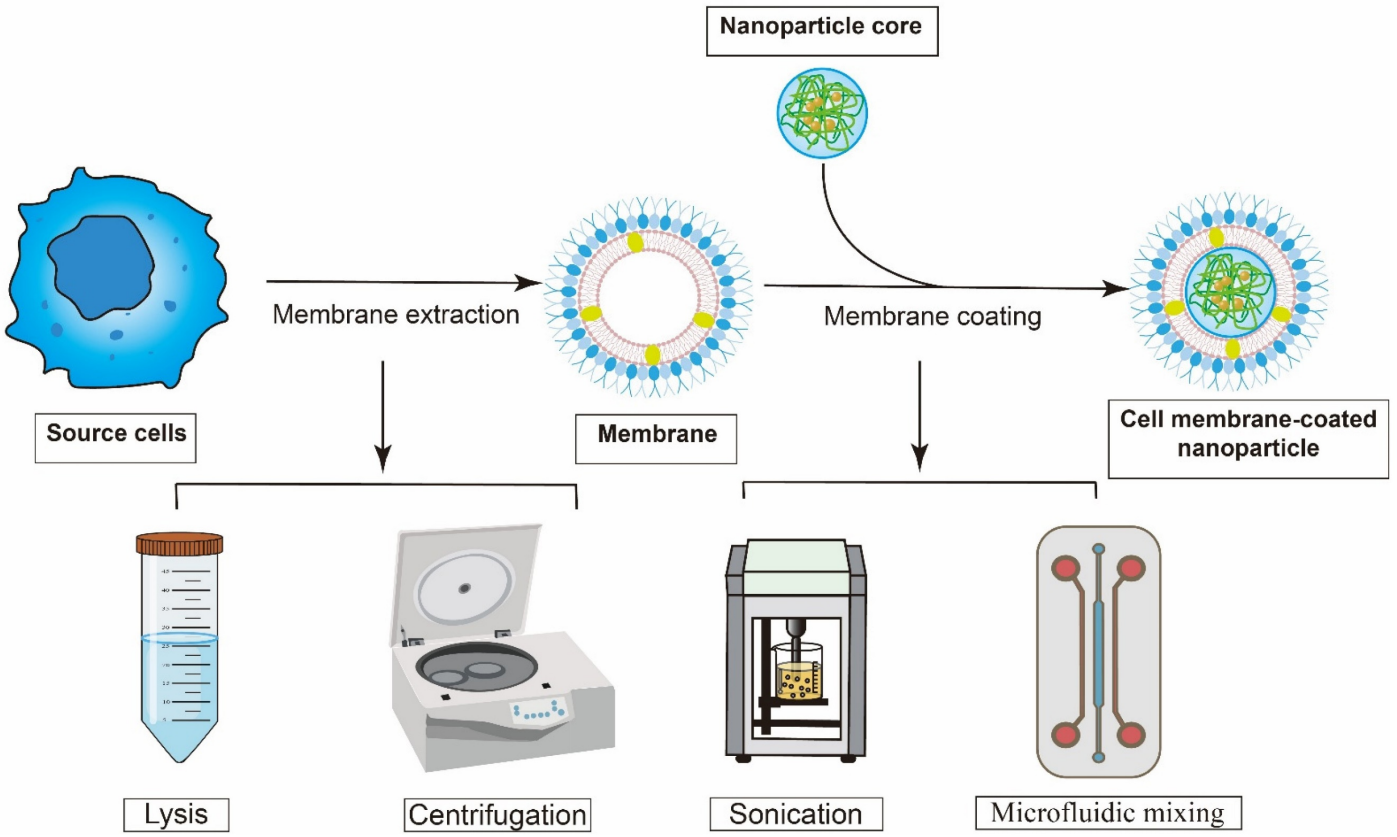
** The basic process of construction of cell membrane-coated tumor vaccine.** Cell membranes were isolated and purified from various source cells using techniques like centrifugation and lysis. Then, these extracted membranes were applied onto the core through ultrasonic agitation or microfluidic mixing.

A typical cell membrane-derived nanovaccine consists of two fundamental elements: an inner synthetic core and an outer envelope composed of naturally derived cell membranes. Accordingly, the fabrication of cell membrane-derived nanovaccines involves two critical steps (Figure 3): the isolation of the source cell membrane and the encapsulation of a synthetic NPs core with the purified membrane 45.

The initial phase involves the extraction and purification of the source cell membrane. For example, tumor cell membrane could be obtained from tumor cell lines, such as 4T1 and B16-F10, or directly from a patient's own tumor cells. These membranes play a crucial role in a wide variety of essential biological functions. Therefore, the extraction process must be gentle. For most eukaryotic cells, the initial membrane isolation involves a series of steps including hypotonic lysis followed by discontinuous sucrose gradient centrifugation to remove intracellular contents 52. For most bacteria, freeze-thaw cycling is a cell lysis technique involving repeated freezing (at -20°C or -80°C) and thawing (at room temperature or 37°C). The formation of ice crystals during freezing mechanically disrupts cellular membranes and internal structures, while this gentle process often helps preserve protein integrity 53. Then add lysozyme to dissolve and digest the extracted cell mixture. To control the process of lysis, sonication has been widely used in laboratory by monitoring the increase in energy input 54.

The second step is the coating. Co-extrusion is a process in which a mixture of purified membranes and nanoparticles is repeatedly passed through a polycarbonate membrane. This method is efficient, however, hard to prepare on a large scale. A significant limitation of this approach is the tendency for filter pores to clog, which reduces production rates and poses a sterility risk 55. Sonication is another method, which uses disruptive force of sonication to induce fusion of membranes and nanoparticles. Although the procedure is technically simple, the outcome is influenced by multiple factors, including sonication amplitude, duration, and temperature 56. Microfluidic mixing is an emerging technique that utilizes tiny channels, measuring from a few micrometers to millimeters, to precisely manage volumes from picoliters to nanoliters during the process 57. The facile fabrication of control chips allows easy scaling from a single mixer to multi-mixer devices, increasing throughput, production, and reproducibility 58. Furthermore, compared to traditional extrusion methods, microfluidics offers superior control, ensuring reproducible outcomes under aseptic conditions.

Successful coating of the NPs core with the cell membrane could be verified by characterizing changes in hydrodynamic diameter and surface charge. In addition to confirming coating success, it is essential to verify that the enveloping membrane maintains both its proper structural orientation and inherent biological activity, as these characteristics are essential for the nanoparticles to achieve optimal performance. Western blotting is another key analytical technique for this purpose, enabling the detection of specific surface proteins on the coated NPs to confirm their presence 59. Flow cytometry functions as a high-throughput analytical technique capable of characterizing large cell populations on an individual cell basis 60. In recent years, high-sensitivity flow cytometry has been introduced to characterize nanoparticles, which could be used to detect intracellular distribution 61. Colocalization analysis is a widely used method for evaluating NPs trafficking and membrane protein interactions. Fluorescence colocalization, the most common technique, involves labeling both NPs and targets with distinct fluorophores. The cross-correlation between the two fluorescence signals is analyzed to characterize NPs behavior 62. Cryogenic transmission electron microscopy (cryo-TEM) is a widely employed technique for imaging biological samples under near-native, hydrated conditions 63. A major strength of cryo-TEM is its ability to determine the 3D structure of nanoparticles through electron tomography. This involves acquiring multiple projection images of the same specimen region at cryogenic temperatures, thereby preserving structural integrity and avoiding drying artifacts 64.

## The emerging cell membrane-coated biomimetic nanovaccines

**Figure 4:**
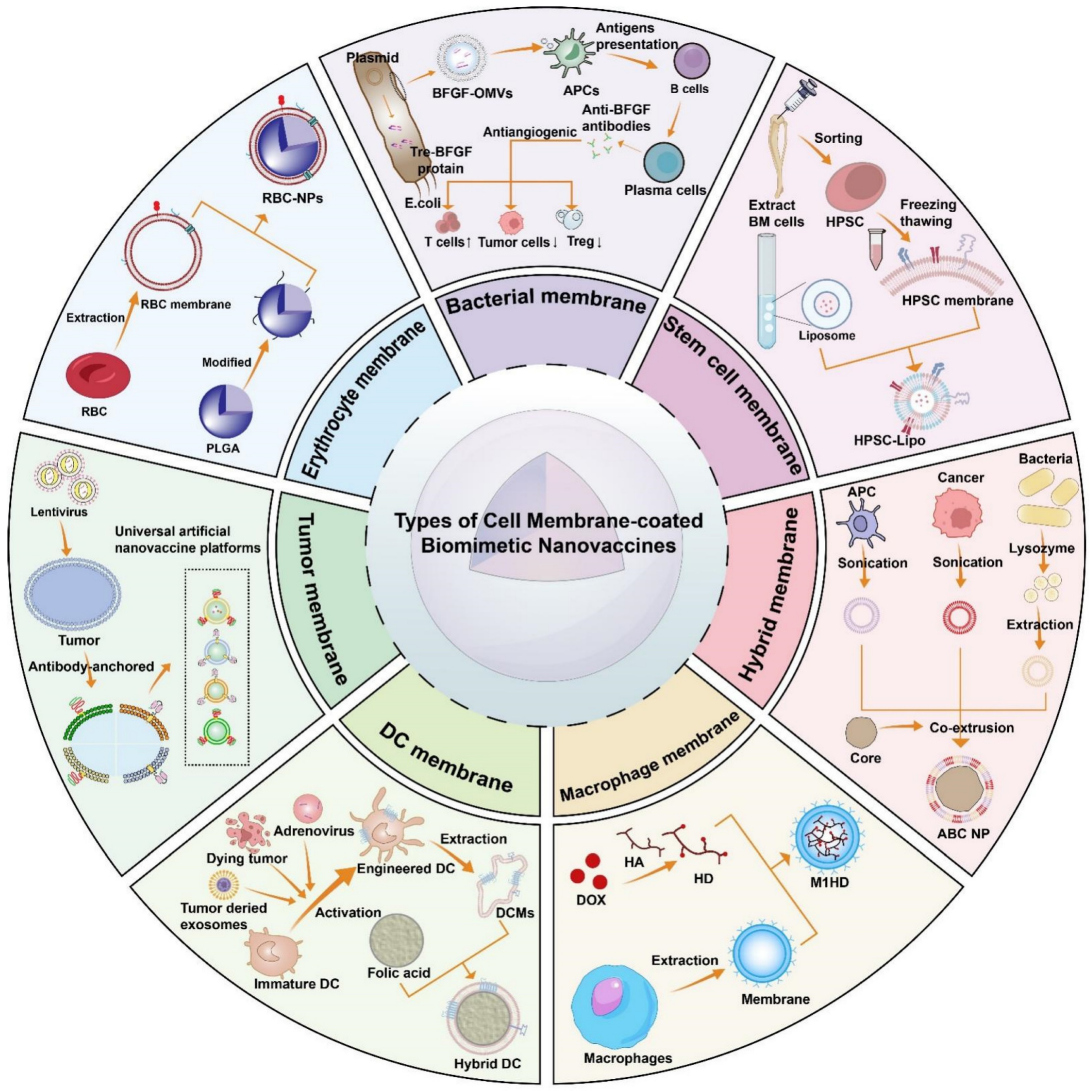
The common types of cell membrane-coated biomimetic nanovaccines and its basic assembly process.

Biomimetic nanovaccines are composed of a core containing specific tumor antigens, adjuvants that enhance the presentation efficiency, and an outer membrane. The different types of cell membrane-coated biomimetic nanovaccines are concluded as follows (Table 2) (Figure 4). Various types of cell membrane-coated biomimetic nanovaccines are characterized by their own sets of advantages and limitations (Table 3).

### Tumor cell membrane

Tumor cell membranes are enriched with a diverse array of source cell-derived tumor antigens, encompassing both tumor-associated antigens (TAAs) and tumor-specific antigens (TSAs), which could elicit tumor-specific immunity 77. Besides, due to central tolerance, the immune system is less likely to erase the tumor cell membrane coated vaccine 91. In this study, the researchers found that the median fluorescence intensity of HeLa cells treated with the membrane coated NPs increased by 3.6 times and that of macrophages decreased by 3 times, compared with NPs 91. There is also a limitation. Protein membranes utilized in vaccine development might contain specific immunosuppressive factors, particularly tumor-associated molecules like programmed death ligand 1 (PD-L1), cytotoxic T-lymphocyte associated protein 4 (CTLA-4), and TIM-3. These components could potentially weaken immune monitoring 92, 93. his indicates that the original cell membranes require comprehensive characterization and thorough screening.

Additionally, vaccines coated with tumor cell membrane are considered to possess a homologous adhesion ability, which offer precise delivery to tumor sites and improve vaccine utilization 76. To achieve robust immune system activation, modifying membrane with specific immunostimulatory molecules could enhance the antigen capture and presentation to APCs 27. However, there still lies striction. The immunosuppressive TME remains a major limiting factor, which partially accounts for the inadequate antitumor effectiveness of biomimetic vaccines 78. Tumors often evade immune attack by suppressing antigen presentation of APCs, thereby diminishing the antitumor efficacy of vaccines 94.

Choosing high immunogenic antigens largely determines the efficiency of tumor, which shows strong affinity with MHC molecules. For example, Senescent tumor cell membrane emerges as highly immunogenic entities, which could trigger efficient anti-tumor immune responses, due to the production of the senescence-associated secretory phenotypes (SASPs) 95. However, certain immunosuppressive components in SASPs could polarize immature myeloid cells toward a suppressive phenotype. This polarization may consequently impair antigen presentation and the functional activation of APCs, potentially leading to immunostimulatory effects 96.

Besides, modifying the membrane with co-stimulatory molecules could enhance the immune response. For example, Li *et al.* 66 developed an antibody-anchored membrane nanovaccine (Nano-AAM/CD40) by incorporating an anti-CD40 single-chain variable fragment (scFv) into a tumor cell membrane, which was subsequently coated onto a NPs core co-loaded with tumor lysates. The cell lysates gave nanovaccine an integration of multiple immunogenic components which could improve immune responses against malignant tumors. Furthermore, to avoid central immune tolerance, an agonist anti-CD40 scFv was anchored to the membrane. This design strategically enhances T cell activation, thereby promoting a more robust and tumor-specific immune response.

**Figure 5:**
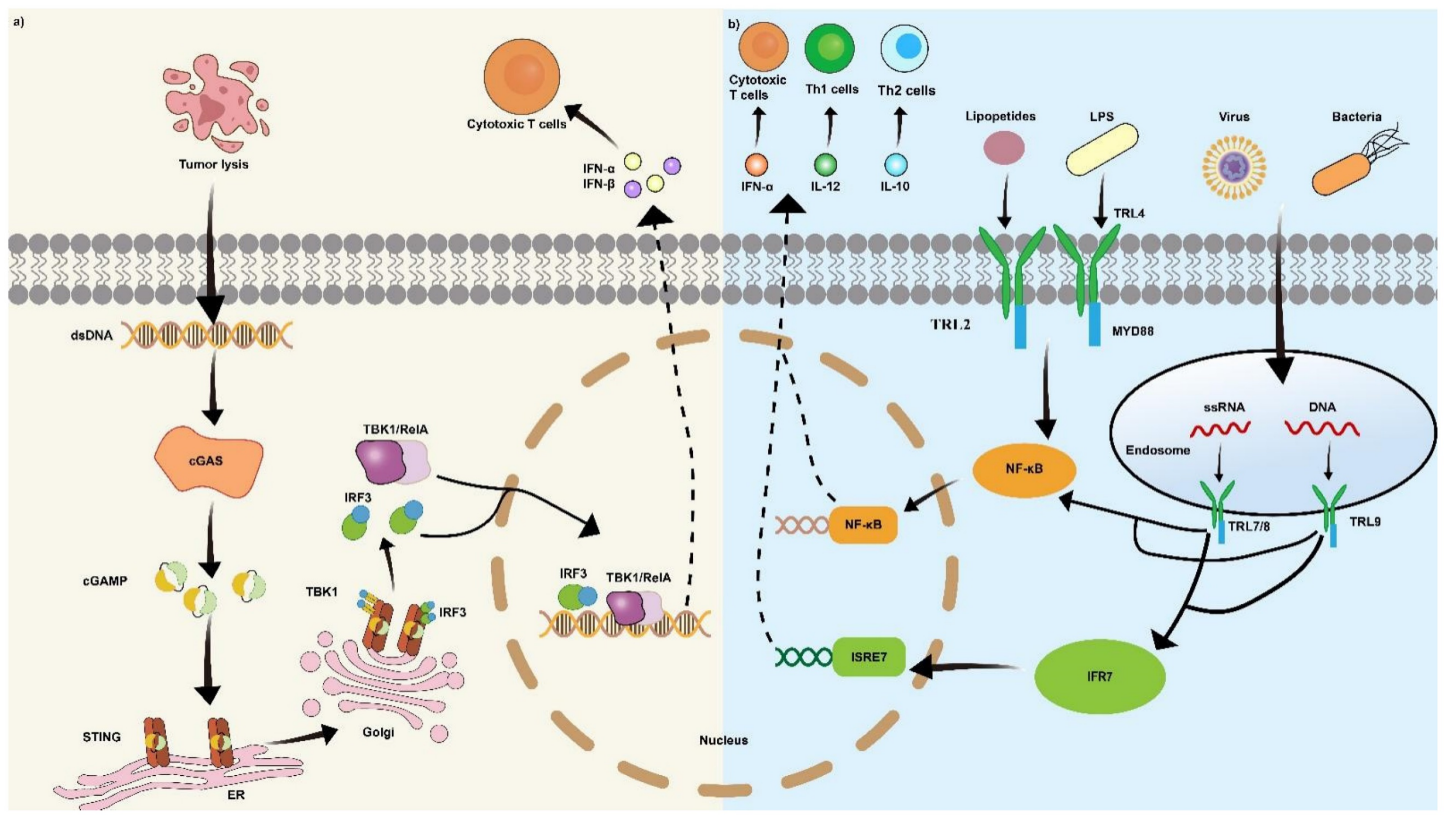
** Major STING, TLR signaling pathways in immune activation. (a)** Upon recognition of cytoplasmic tumor-derived DNA, cGAS generates the second messenger cGAMP, a natural STING ligand. cGAMP binding induces STING activation and conformational rearrangement, initiating a TBK1-IRF-3 signaling cascade that drives type I interferon expression. **(b)** The type of TLR response is dictated by signaling pathways activated by a specific TLR and its adaptor proteins. Myeloid differentiation primary response protein 88 (MyD88) serves as a universal adaptor for nearly all TLRs except TLR3. Upon activation, MyD88 initiates a signaling cascade that ultimately activates the NF-κB pathway, resulting in the production of proinflammatory cytokines. In addition, TLR7 and TLR9 utilize MyD88 to activate interferon-regulatory factors (IRFs) 3 and 7 via TRAF3, thereby inducing a type I interferon response.

An alternative strategy for immune activation involves the delivery of stimulator of interferon genes (STING) agonists. STING is a protein located in the endoplasmic reticulum. It acts as a sensor for cytosolic DNA, which can come from infections, cellular damage, or cancer. In the cytosol, DNA binds to cyclic GMP-AMP synthase (cGAS) to produce cyclic GMP-AMP (cGAMP). The cGAMP molecule activates STING, thereby inducing cellular production of IFNs. As pivotal cytokines, type I IFNs bridge innate and adaptive immunity to stimulate potent antitumor and antiviral responses 97. However, the limited way of STING agonists' delivery restricts its clinical uses. Most of them can only be injected intratumorally, while systemic administration shows significant toxicity 98. Tumor vaccine emerged as the carrier to deliver STING agonists with its specific targeting ability. In the study of Gou *et al.* 65, the team developed a peptide CBP-12 expressed biomimetic tumor cell membrane-coated NPs to target Clec9a DCs and deliver STING agonists. The researchers surprisingly found that the STING agonist stimulated Clec9a DCs more efficiently than tumor cells. Administration of a STING agonist activates the cGAS-STING pathway, triggering type I IFNs release. The released IFNs stimulates DCs activation and antigen cross-presentation, thereby priming T cells (Figure 5a).

An additional benefit of nanovaccines is their capacity to facilitate the delivery of adjuvants. Adjuvants are molecule that could enhance the efficiency of vaccine through specific ways, such as promoting antigen presentation and inducing immune cell maturation 51. Toll-like receptor (TLR) agonists have demonstrated efficacy in enhancing the functional maturation and antigen-presenting capacity of DCs. Using the TLR agonists, DCs are induced to secrete Th1-specific cytokines, such as IL-12 and TNF-α which boost vaccine adaptive immune responses (Figure 5b) 99. For example, monophosphoryl lipid A (MPLA), a TLR-4 agonist 100, and miquimod (IMQ), a clinically approved TLR-7 agonist 101, are the most widely used TLR agonists.

B lymphocytes, due to lack of costimulatory molecules is not being much noticed in tumor therapy. However, Yan's team developed a triple negative breast tumor (TNBC) membrane coated, CpG and aCD40 conjugated vaccine 102. The nanovaccine could activate B cells, leading to secretion of tumor-associated antibodies and improving the ability of B cells to present antigens, which in turn activates T cells. This exciting strategy might pave the way for ushering the era of B cell-based immunotherapy.

However, the time-consuming procedure manufacturing process has significantly constrained the therapeutic window and hindered the broad clinical adoption of tumor vaccines. To address this limitation, a research team developed an AECM strategy, which using IFN-γ to stimulate antigen presentation 103. The resulting tumor cell membranes could provide adequate tumor responsive antigens especially pMHC-I compared to vaccines derived from whole tumor cell lysates. In tumor environment, DCs often lack MHC-I which significant limit the capacity of antigen presentation. However, MHC-I-deficient dendritic cells can acquire pMHC-I from AECM through a process known as cross-dressing. By directly utilizing these acquired pMHC-I, the DCs are able to prime T cells effectively, thereby initiating a robust antitumor response.

### Dendritic cell membrane

Mature DCs exhibit high levels of the MHC class I antigen complex, costimulatory molecules such as CD80 and CD86, as well as chemokine receptors including ICAM-1 and CCR-7. Therefore, DCs membrane coated vaccine has a better ability to target the lymph nodes 79. Additionally, the DCs membrane-coated vaccine could promote efficiency in antigen uptake, processing and presenting, which significantly strengthen the activation of immune system. The vaccine utilized membrane proteins (such as MHC, CD86, and CD40) to mimic DCs' antigen presentation ability and releases IL-2 in a paracrine way. This could activate T cells and trigger a strong antitumor immune response without being affected by immunosuppressive tumor-associated macrophages (TAM) and physiological barriers during cellular migration and antigen presentation. 104.

Vaccine could reshape the immune TME, by promoting tumor-infiltrating lymphocytes to improve therapeutic effect. Wang *et al.* developed a vaccine coated with dendritic membrane stimulated by H22 mouse liver tumor 21. The nanovaccine was engineered with a mesoporous silica core co-loaded with silicon phthalocyanine dichloride (SiPcCl₂) as a photosensitizer and Fe (III)-captopril complexes to modulate neutrophil polarization. The Fe (III)-captopril component suppresses tumor growth by reprogramming pro-tumoral N2 neutrophils to the anti-tumoral N1 phenotype. This phenotypic shift is believed to inhibit angiogenesis, particularly at the invasive margin of hepatocellular carcinoma (HCC), by altering the functional state of peritumorally infiltrated neutrophils. More importantly, it could target tumor to enhance ICD treatment by driving T cell immune response, which means convert tumor immune “cold” state into “hot”.

There are studies report that live cell-based vaccines have possible disadvantage such as their short shelf life, vulnerable in immunosuppressive conditions, and insufficient lymph node delivery*.* To solve this shortage, utilizing modified membrane is an emerging strategy 80. In the study of Wang *et al.* 67, they created a nanovaccine in which radiotherapy-induced immunogenic antigens and tumor-derived exosomes (TEXs) were mixed with the extracted and cultivated *in vitro* bone marrow-derived dendritic cells (BMDCs) to deliver whole-tumor antigens. The complex retained mature DCs-derived costimulatory molecules (CD80/CD86), MHC-I antigen complexes, and the chemokine receptor CCR7, thereby significantly enhancing lymph node targeting and antigen presentation efficiency.

A recent study has proposed a framework for advancing the clinical translation of cancer vaccines through the development of the ASPIRE strategy 105. ASPIRE is characterized by the directional presentation of specific antigenic epitopes via MHC-I molecules and the co-delivery of an anti-PD-1 antibody and B7 costimulatory molecules. Unlike conventional vaccines that require uptake and processing by APCs, ASPIRE directly presents neoantigens to CD8^+^ T cells, thereby effectively stimulating cytotoxic T lymphocyte (CTL) responses without the need of DC maturation. Furthermore, the incorporation of anti-PD-1 functions synergistically with CD28/B7 co-stimulation to sustain prolonged CTL activity, enhancing the durability of the immune response.

### Erythrocyte membrane

Recent studies suggested a common phenomenon in NPs clearance called accelerated blood clearance (ABC). The ABC phenomenon induced is characterized by rapid clearance of subsequent doses from circulation and markedly enhanced liver accumulation 106, 107. Compared with other cell membrane, vaccine coated with erythrocyte membrane has a unique advantage of longer circulation time, due to the CD47 on the membrane. This molecular present a signal of “don't eat me”, which could resist the clearance and longer the circulation time 81. Furthermore, red blood cell membranes contain other membrane proteins such as C8 binding protein (C8bp), homologous restriction protein (HRP), decay accelerating factor (DAF), membrane cofactor protein (MCP), complement receptor 1 (CR1), and CD59, which collectively contribute to the against system-mediated immune attack. However, simple coating of red blood cell membranes lacks specific targeting capabilities 83. The blood groups of both donor and recipient must be determined for compatibility, which limited its use 82.

Adjusting specific protein or other substance to modify erythrocyte membrane is an important method to extend utilization and increase efficiency. For example, Guo *et al.* design a platform which used erythrocyte membrane to envelope PLGA-NPs 68. In the described delivery system, the functionalized membrane enables targeted binding to mannose receptors. As carbohydrate-binding receptors expressed on macrophages, DCs, and nonvascular endothelium, mannose receptors contribute significantly to antigen recognition and the promotion of anti-tumor immunity. Additionally, using the membrane the insertion capacity to add the lipid-like adjuvant of MPLA, which could enhance presentation of antigen in APCs. Also, adding the melanoma-associated antigenic peptides hgp10025-33 to target the tumor. This complex shows significant suppression in tumor prevention, growth, and metastasis.

Erythrocyte membrane enveloped NPs, significantly smaller than native red blood cells, could escape from the blood vessels of tumors and penetrate into the inner regions of solid tumors. Gao *et al.* developed a biomimetic nanoplatform by coating NPs with a red blood cell membrane and encapsulating perfluorocarbon (PFC) 108, a widely employed artificial blood substitute. This system could improve hypoxia in solid tumor which could lead to resistance of tumors to radiotherapy. Following intravenous injection, the NPs extravasate from the tumor vasculature and diffuse into the tumor parenchyma, significantly enhancing overall tumor oxygenation and thereby increasing radiosensitivity.

### Macrophage cell membrane

Similar with erythrocyte membrane, macrophage cell membrane exhibits a series of self-markers such as CD47, which gives its cargo a longer circulation time without being clearance 109. As a membrane of natural immune cell, it has the capability to sense chemotactic cues to target to tumor efficiently 84. Additionally, studies report that macrophage could get into the tumors lacking blood vessels which are inaccessible for tradition deliver platform 85. Blood-brain barrier (BBB) functions is a highly selective barrier that limits the penetration of the most drugs and nanoparticles into the central nervous system (CNS). However, macrophage cell membrane demonstrates its unique ability to penetrate it through transcytosis or temporarily disrupt integrity of BBB by releasing inflammatory signals 86. Despite macrophages have great potential as novel drug delivery vehicles, it confronts a series of challenges. The ability of macrophages to migrate to tumors needs to be thoroughly verified, as macrophages often become trapped in the lungs after being injected into the bloodstream, and then move to the liver and spleen 85.

Macrophage's function diverges by phenotype: M1 macrophages exert antitumor effects, while M2 macrophages support tumor progression. Hence, M1 macrophages target tumor cells is a common strategy. Yang *et al.* reported a M1 macrophage membrane-coated platform enveloping with DOX, chemotherapeutic drugs, and shRNA-Ptpn2, the target antigen of melanoma 75. The complex was phagocytosed by M1 macrophage which improved NPs evasion of the reticuloendothelial system (RES) and promoted their accumulation at tumor sites. The NPs downregulate Ptpn2 gene to trigger the activation of CD8^+^T cells and polarization of M1 macrophages. Another example is a membrane-camouflaged Bacillus Calmette-Guérin (BCG) invented by Zhang's team 110. In the study, researchers utilized macrophage membranes to coat BCG to induce trained immunity in tumor-associated macrophages. The membranes could protect BCG from immune system elimination and facilitate tumor targeting capability in mouse model of Lewis lung tumor (LLC).

Tumor-associated macrophages (TAMs) largely exhibit anti-inflammatory M2 polarization, which induce tumor angiogenesis, invasion, metastasis, and immune evasion 111. Thus, Targeted TAMs can induce a more durable antitumor immune response by alleviating immunosuppression and creating an environment conducive to T-cell activity. In Zhao's study, the team developed a macrophage cell membrane-coated PLGA nanoparticle loading TMP195, a drug reprogramming TAMs 112. The NPs function to repolarize TAMs toward the M1 phenotype, thereby restoring their antitumor and pro-inflammatory functions. Emerging evidence suggests that reprogramming tumor-associated macrophages from the immunosuppressive M2 phenotype to the pro-inflammatory M1 phenotype represents a promising strategy to inhibit localized tumor recurrence and suppress distant metastasis 113.

### Stem cell membrane

Stem cells encompass several major types, including induced pluripotent stem cells (iPSCs), hematopoietic stem and progenitor cells (HSPCs), and mesenchymal stem cells (MSCs) 114. Despite residing in different tissues, all stem cells share some common characteristics. Due to its low immunogenicity, stem cell membrane-coated vaccines could avoid immunosurveillance, and has a extend circulation time. Besides, most type of stem cells possess an inherent tropism that enables tumor homing 115.

MSCs are capable of traversing biological barriers, which are tailored for targeted delivery to spinal cord, brain tumor 87. However, A key challenge with intravenous MSC delivery is their accumulation in the lungs and liver, thereby limiting insufficient concentrations at the targeted tissues. Through genetic engineering to overexpress chemokine receptors, the tumor-homing efficiency of MSCs can be significantly improved 50. Wang's team developed a MSC membrane coated nanoplatform loading αPD-L1 to treat Glioblastoma multiforme (GBM). Based on analysis of the radiation-mediated inflammatory microenvironment in GBM, CC chemokine receptor 2 (CCR2) was selected for overexpression on MSCs to enhance their tumor targeting 116.

HSPC-mediated bone marrow targeting delivery drugs is a promising strategy in the treatment of leukemia. Li *et al.* developed biomimetic vesicles for leukemia treatment by fusing HSPC membranes with liposomes (HSPC-liposomes) 71. Targeted drug delivery to bone marrow represents a promising therapeutic strategy. To enhance targeting specificity, the researchers leveraged CD44, an adhesion molecule expressed on HSPCs, to enable recognition of hyaluronic acid (HA). Notably, HA serves as a canonical ligand for CD44 and is enriched in the bone marrow, thereby providing a specific anchoring mechanism.

iPSCs could express different shared types of the tumor oncofetal antigens, which makes iPSCs a potentially robust source of antigenic material for prophylactic anti-tumor vaccination for many tumors 88. Krishnan *et al.* 70 developed a vaccine coated with iPSCs membrane, which programmed from mouse tail tip fibroblasts. The vaccine had a biodegradable polymeric core loaded with CpG oligodeoxynucleotide 1826, a TLR9 agonist. The vaccine demonstrated efficacy in promoting DCs maturation and activating T-cell responses on five different murine tumor models which show its broad specificity.

### Bacterial outer membrane vesicles

Bacterial outer membrane vesicles (OMVs) are naturally released through the controlled blebbing of the outer membrane in Gram-negative bacteria, a process triggered by perturbations of the cell envelope 117. OMVs are rich in pathogen-associated molecular patterns (PAMPs) which could stimulate strong antitumor adaptive immunity 89. Additionally, OMVs has a unique cargo transport capability. It could envelop molecules with poor solubility to prevent them being degraded and concentrate the dose above the minimal effective concentration which make OMVs specially tailored for long distances delivery. Besides, OMVs could stimulate the immune system due to its richness of pattern recognition receptors (PRRs). The cargo could be delivered directly into the immune cell to regulate immunomodulatory. For example, in Huang's study 73, the team presented full length of BFGF molecule of mice onto the OMVs by using genetic modification, which significantly induce the production of anti-BFGF autoantibodies and inhibited angiogenesis in tumor tissues to increase tumor cell apoptosis.

The presence of bacteria within many tumors enables microenvironmental remodeling that protects cancer cells from immune clearance 118. In Shen's study 119, the team develop a nanoplatform coated with OMVs secreted by Fusobacterium nucleatum (FMVs), loading antibacterial drug cinnamaldehyde (CA). Core contain polyethyleneimine-ferrocene (Fc-PEI) to promote dual ferroptosis in intratumoral bacteria and tumor. Thus, breast cancer treatment can be enhanced by relieving immunosuppression and activating tumor ICD.

However, there are more and more evidence show that maturation-induced uptake obstruction (MUO) occurred when DCs uptake OMVs, which lead to a limited uptake capacity 90. DCs internalize OMVs via specific binding of LPS on the OMVs surface to TLR4 expressed on DCs. However, the rapid maturation of DCs induced by immunostimulatory components in OMVs attenuates the subsequent interaction between LPS and TLR4. This phenomenon attenuates sustained antigen presentation, resulting in a limited antigen-specific immune response. To minimize the side effect of MUO, in Liang's study 90, the team load αDEC205 antibody to OMVs. DEC205 represents a surface molecule whose expression is upregulated downstream of TLR pathway activation in mature DCs. The study suggests that this targeting antibody recognition could prolong the uptake when the DCs are mature, which show a boost in antigen presentation and T cells activation.

However, OMVs-based nanocarriers possess inherent limitations that constrain their further development and clinical advancement. For example, OMVs contain large amounts of proteins, polysaccharides, lipids and trace amounts of nucleic acids from bacteria, which poses significant challenges for quality control in vaccine manufacturing 120. There is an urgent need for suitable purification strategies compatible with large-scale industrial production. One potential approach involves chromatographic separation based on the size of outer membrane vesicles.

### Hybrid membrane

Scientists mixed two or more type of different cell membrane to combine all its advantage and compensate for each shortcoming 121. For example, Ren *et al.* developed an “ABC” ternary membrane vaccine platform, which integrates membranes from three distinct sources: antigen-presenting mature DCs (“A”), bacterial E. Coli (“B”), and tumor cells (“C”) 74. In the combination, DCs membrane could not only promote interaction between vaccine and immune cells, but also improve the accumulation in lymph nodes. Rich in PAMPs, cytoplasmic membranes could enhance the immune system as adjuvants. While, hybrid membrane-based vaccines deliver a comprehensive antigen repertoire comprising both TAAs and TSAs from tumor cells, alongside endogenous MHC molecules derived from DCs. This integrated antigen presentation promotes a more diverse T-cell repertoire 122. The dual targeting of lymph nodes and tumors could reshape immunosuppressive microenvironment and lead T cells infiltration 123.

Hybrid membrane technology can extend the therapeutic potential of tumor vaccines, particularly in addressing diverse tumor complications. Chen reported a dual-antigen-displaying nanovaccines 124. Antigens were hybrid with ovalbumin (OVA) on tumor membrane and spike (S) glycoprotein on induced T cell membrane. This strategy demonstrates dual efficacy in preventing SARS-CoV-2-related complications and exerting antitumor effects.

The clinical transformation of hybrid membrane tumor vaccines lags behind, largely due to the complexity of the manufacturing process and the inherent defects of various membranes. For example, when destruction of OMVs membrane and tumor membrane based nanovaccine 120, immunosuppressive factors of the tumor cell membrane and complicated components of OMVs significant limit its efficiency. Furthermore, there remains an urgent need for scalable, sterile manufacturing processes suitable for the industrial production of OMV-based nanocarriers.

## The combination of biomimetic nanovaccines with other anti-tumor therapies

**Figure 6:**
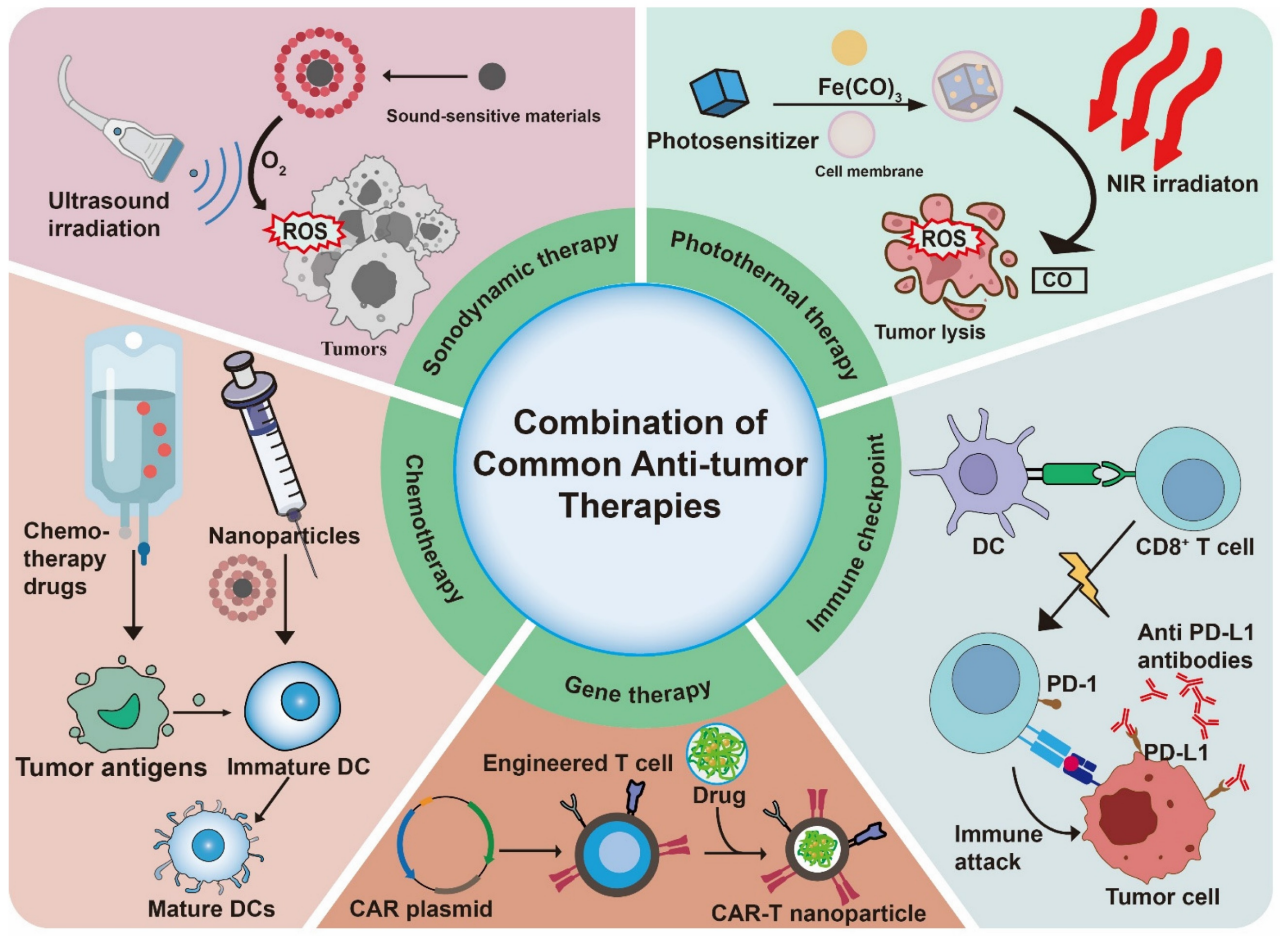
The combination of biomimetic nanovaccines with common anti-tumor therapies.

Biomimetic nanovaccine has been applied to combine with common antitumor therapies (Table 4) (Figure 6). They are two major reasons for the combination. The first is the powerful load and carrying capacity of nanoparticles, which offer a targeting ability for other therapy delivering drug 103. Secondly, other antitumor therapy could induce ICD which is a key approach in developing tumor vaccines because it directly releases a wide variety of tumor-associated antigens. This significantly boosts the availability of antigens and stimulates strong, durable antitumor immune responses, ultimately improving the effectiveness of the vaccine 125.

### Nanovaccines and chemotherapy

Chemotherapy is one of the most widely used anti-tumor therapies. However, due to a lack of targeting ability, the extensive uses of chemotherapeutics could lead to severe systemic toxicity and immune resistance, which significantly limited its uses 136. Research indicates that combining nanotechnology with chemotherapy may eliminate the side effects especially systemic toxicity associated with chemotherapy 126. Nanovaccine usually contains a strong and targeting drug delivery system which could load chemotherapeutics. And chemotherapeutics could induce ICD to enhance the efficacy of nanovaccine. In the study of Wang *et al.*, they combined tumor cell membrane coated vaccine with DOX to treat fibrosarcoma 3. The team constructed a platform based on fibrosarcoma cell membranes coated PLGA loaded with the STING pathway activator 2,3-cGAMP. After vaccine ejection, the intravenous injection of low-dose DOX induced ICD. Critically, the incorporation of DOX effectively counteracted vaccine-induced myeloid-derived suppressor cell (MDSC) upregulation, thereby reversing its associated immunosuppressive effects.

A considerable proportion of patients eligible for personalized tumor vaccination have often received prior chemotherapy. Research has shown that chemotherapy may improve the immune environment within tumors, promoting a more activated immune response. However, chemotherapy-induced systemic toxicity frequently leads to immunosuppression 127. Therefore, a critical need exists to evaluate the potential influence of preoperative chemotherapy on subsequent vaccine efficacy. In Chen's study, researchers investigated nano-formulated liposomal DOX as preoperative chemotherapy on the efficacy autologous tumor cell membrane antigen-based vaccines 137. This study suggests that preoperative chemotherapy could not only modify the immune microenvironment toward immune activation, but also upregulate immune-related molecule expression, boosting the immune response when used in vaccines. Similarly, in Bao's study, the team constructed an integrated therapeutic system comprising a DOX-releasing prodrug (R-TD) and a nanovaccine platform (RBC-NPs), which acted synergistically to suppress solid tumor growth 138. This combinatorial strategy enhanced anti-tumor immunity by augmenting DCs and cytotoxic T lymphocyte (CTL) infiltration in lymph nodes and elevating cytokine secretion, while concurrently attenuating immunosuppression through suppression of regulatory T cell (Treg) activity. The strategic integration of chemotherapy and immunotherapy represents a promising approach for achieving superior anti-tumor efficacy compared to monotherapy.

### Nanovaccines and immune checkpoint therapy

Immune checkpoint therapy is another emerging anti-tumor strategy. Lots of molecules contribute to the regulation of immune cell, such as PD-1/PD-L1 and CTLA4/CD86. Tumor vaccine could carry co-stimulatory molecules to enhance its efficiency. Lu *et al.* 129 developed an OMV-based nano-vaccine called APSE delivering neoantigens, adjuvants, and αPD-L1 antibodies to DCs, which could enhance antigen presentation of DCs. Additionally, compared to the current systemic administration of anti-PD-L1 therapy *in vivo*, this strategy reduced the αPD-L1 antibody dose by 10 times with lower toxicity and side effects. CD47, commonly overexpressed on various tumor cell surfaces, binds to signal-regulatory protein alpha (SIRPα) on myeloid cells to transmit a “don't eat me” signal, thereby evading immune clearance. Blockade of the CD47-SIRPα immune checkpoint axis enhances the phagocytic clearance of tumor cells by APCs. Liu *et al.* 128 employed CRISPR-Cas9 technology to knock out the CD47-SIRPα phagocytosis checkpoint *in vitro*, then isolated membranes from these CD47KO/CRT dual-bioengineered tumor cells to coat NPs cores loaded with CpG adjuvants. The vaccine elicited an “eat me” signal that promoted antigen uptake and presentation by APCs, leading to the activation of tumor-specific CD8^+^ T cells and subsequent tumor cell killing.

Despite the widespread clinical adoption of immune checkpoint inhibitors (ICIs) in recent decades, their efficacy remains limited, largely attributable to the immunosuppressive TME. Evidence suggests that ICD remodels the tumor immune microenvironment, thereby enhancing the efficacy of ICI therapy. Li *et al.* engineered a biomimetic NPs that amplifies the ICD effect through ROS-mediated signaling 139. The NPs system encapsulates the ICD inducers epirubicin (EPI), glucose oxidase (Gox) and hemin within a ZIF-8 framework, which is subsequently coated with a tumor cell membrane. Upon cellular internalization of the vaccine, a Fenton reaction is triggered that enhances ROS production and elevates endoplasmic reticulum (ER) stress, thereby amplifying the ICD effect. This mechanism promotes the release of tumor-associated antigens and damage-associated molecular patterns (DAMPs), leading to activation of the tumor immune microenvironment.

### Nanovaccines and photothermal therapy

Photothermal therapy (PTT) uses photothermal agents to absorb near-infrared (NIR) laser, converting it into localized heat to selectively kill tumor cells 140. As a minimally invasive modality and fewer side-effect, PTT is suitable for various solid tumors. 141. Coupled with vaccine, PTT could improve the precision of treatment, minimizing off-target damage. Shi *et al.* 130 constructed a zinc phosphate-based NPs platform (LDC@ZnP NPs) that integrates DCs membranes with two functional cargos: the colon carcinoma (MC38)-specific antigen Adpgk and the natural photosensitizer melanin. Following NIR irradiation, the melanin component demonstrated efficient photothermal conversion while maintaining excellent biocompatibility with immune cells. When loaded in vaccine, the melanin displayed better photothermal effects than free melanin probably due to the encapsulation of nanovaccine.

An additional benefit of the integration of tumor vaccines with photothermal therapy is that PTT has the potential to release endogenous tumor antigens, thereby enhancing the immunogenicity of the vaccine 131. Yin *et al.* 142 engineered a tumor vaccine platform by coating Prussian blue-carbon monoxide NPs (PB-CO NPs) with 4T1 mammary tumor cell membranes (4T1-PB-CO NPs), and combined it with anti-IL-10 treatment to maximize therapeutic efficacy. Upon NIR irradiation, 4T1-PB-CO NPs released carbon monoxide (CO), inducing the generation of endogenous TAAs that subsequently activated a CD8^+^ T-cell response. By integrating exogenous TAAs presented on the 4T1 cell membrane with anti-IL-10 therapy, this dual-pronged vaccine strategy demonstrates enhanced capacity to reverse immunosuppression in the TME and potentiate anti-tumor immunity.

### Nanovaccines and sonodynamic therapy

Sonodynamic therapy (SDT) could produce ROS in the presence of O_2_ through ultrasound activation of sonosensitizers 143. Compared with NIR, ultrasound has a better tissue-penetrating ability, which could precisely induce apoptosis in pathological cells while preserving the integrity of surrounding normal tissues 132. Recent studies indicate that ROS serve as a potent immunological trigger by enhancing proteasomal degradation of proteins and facilitating MHC-I antigen presentation. This mechanism suggests that well-designed sonosensitizers could significantly improve vaccine efficacy 133. Sun *et al.* 144 co-encapsulated the sonosensitizer chlorin e6 (Ce6) and engineered tumor cell-derived mRNAs within PLGA NPs functionalized with tumor membrane proteins. Under ultrasound irradiation, Ce6 facilitates endosomal escape of the mRNA cargo, thereby enhancing antigen presentation efficiency. Chen *et al.* reported a ultrasound-activatable *in situ* vaccine loading DNA methyltransferase inhibitor zebularine (Zeb) and sonosensitizer hematoporphyrin monomethyl ether (HMME), which were coated with hybrid membrane derived from a thylakoid (TK) and platelet (PLT) membrane 145. Researchers employ ultrasound strategy to trigger immunogenic tumor death to promote antigen release for *in situ* tumor vaccination. The nanovesicle showed promise in enhancing antigen cross-presentation by DCs and activating T cells, while also targeting both primary tumors and potential metastatic sites.

### Nanovaccines and gene therapy

Gene therapies are rapidly emerging as a critical component of the therapeutic arsenal for diverse inherited and acquired human diseases, including inherited immune deficiencies, hemophilia, ophthalmic and neurodegenerative disorders, as well as hematological malignancies 146. NPs are increasingly utilized in gene therapy due to their unique advantages, including their ability to deliver drugs and enhance the effectiveness of anti-tumor treatments 147.

Chimeric antigen receptors (CARs), representing a prominent form of adoptive cell therapy, are artificially engineered fusion proteins expressed on T cells. These modular receptors consist of an extracellular antigen-binding domain linked to diverse intracellular signaling modules. Upon recognition of TAAs presented on malignant cells, CAR-T cells undergo activation and clonal expansion, culminating in cytokine release and targeted elimination of antigen-expressing tumor cells. While CAR-T therapy has achieved remarkable success in hematological malignancies, its efficacy against solid tumors remains limited, primarily due to the immunosuppressive TME, poor T cell persistence, and a scarcity of truly tumor-specific antigen targets 148. Consequently, researchers are integrating nanotechnology with CAR-T cell therapy as an innovative strategy to overcome the challenges of solid tumor treatment 135. Current studies reported NPs are used to deliver mRNA to generate CAR-T cells *in vivo* 149. Besides, tumor membrane coated nanoparticles could deliver molecular to block immunosuppression of TME, thereby rescuing hypofunctional CAR-T cells 150.

Yamen *et al.* engineered a biomimetic nanoplatform by encapsulating cisplatin loaded PLGA NPs and coating them with CAR-T membranes derived from genetically modified human Jurkat T cells, creating a targeted therapeutic system for HER2-positive lung carcinoma 134. The novel CAR-T-MNPs were fabricated by coating PLGA NPs with anti-HER2 scFv-expressing membranes isolated from genetically engineered Jurkat T cells. This design aims to prolong systemic circulation and enhance tumor-specific accumulation, while simultaneously reducing nonspecific clearance. Experimental results indicated that the biomimetic NPs platform exhibited sustained drug release over 21 days and demonstrated suitability for intravenous administration based on its optimal size, favorable biocompatibility, and robust stability. However, before being applied in clinical practice, these innovative nano-CAR-T therapeutic platforms need to be assessed to confirm their effectiveness and safety.

## Challenges and future perspectives

In recent years, artificial intelligence (AI) has rapidly emerged as a transformative force in tumor vaccine development, significantly accelerating the development of innovative tumor vaccines. From predicting patient-specific antigens, discovering adjuvants 151 to guiding vaccine formulation and supporting clinical trials, AI has the potential to enable truly personalized tumor vaccination strategies 152. Deep learning has been applied to predict MHC-I and MHC-II binding and ligands. NeoDisc was developed to assess tumor heterogeneity along with antigen processing and presentation functionality, demonstrating superior performance in neoantigen prioritization 153. A major obstacle, however, is the lack of sufficiently large, standardized datasets that contain high-quality T-cell response data and can distinguish between immunogenicity and antigenicity 154. AI in vaccine manufacturing needs to be more deeply involved. Zhou's team design a nanovaccine to deliver mRNA antigen and cGAMP for STING activation using machine learning 155. The team employed an AI algorithm to rank the key characteristics of nanoparticles in RNA transcription, then optimizing the structural parameters of nanoparticles for high efficiency. This demonstrates that AI's capabilities in manufacturing go far beyond mere protein screening.

As fundamental research advances, an increasing number of nanovaccines are entering clinical trials. For example, the mRNA-4157 of Moderna has recently entered the phase 2b trial and has shown positive and clinically meaningful results. 156. The treatment method of mRNA-4157 makes use of an mRNA construct that has a lipid nanoparticle formulation, within which are encoded as many as 34 new antigens. After being administered, these mRNA sequences will go through endogenous translation, and then trigger the pathways of antigen processing and presentation. Clinical data demonstrate that the combination of mRNA-4157 with pembrolizumab significantly reduced the risk of recurrence or death compared to pembrolizumab monotherapy, while maintaining a clinically manageable safety profile. This study indicates that combining an mRNA-based individualized neoantigen therapy with PD-1 blockade enhances clinical efficacy in melanoma compared to PD-1 inhibitor monotherapy.

Several challenges still remain for the further development of nanovaccine therapies. GMP is a term recognized worldwide for the control and management of manufacturing and quality control testing of foods, pharmaceutical products and medical devices 157. Meeting GMP standards is a prerequisite for the commercial production of vaccines and other medical products 158. Compared with the process in lab, GMP require safer source of cells, easier scale-up methods and a satisfied yield of the purification process 159. GMP encompasses not only the processes but also the manufacturing environment and the equipment utilized. However, biomimetic cell membrane-coated nanovaccines may hard to meet GMP standard under the current technological conditions.

For membrane extraction, a large part of membrane is extracted from autologous or allogeneic tumor tissues which may contain slight individual differences and tumor heterogeneity. This heterogeneity poses a substantial challenge for isolating and characterizing pure cellular populations. Proteomics is emerging as a valuable tool, enabling the identification of specific targets for affinity-based isolation and facilitating comparative analysis across distinct subpopulations 160. To overcome this obstacle, the establishment of a standardized donor cell bank or the utilization of immortalized cell lines could unify the source of cell membranes, which considerably reduces the risk of sterility loss and potential contamination 161. An alternative strategy to overcome antigen heterogeneity involves targeting surface antigens hat are broadly expressed across tumor cell populations. iPSCs share functional antigenic profiles with tumor cells. Therefore, the analysis of iPSC membranes offers a pathway for identifying shared antigens, which in turn could streamline vaccine manufacturing 162.

The stability of nanovaccines can be impacted by their storage and transportation, potentially diminishing their effectiveness. This necessitates improvements in cold chain management and the innovation of new strategies to guarantee the prompt and safe delivery 163. Besides, risk assessment concerning equipment accuracy and contamination must be conducted throughout the entire process, from material preparation to final production 164. Hence, the implementation of modular and adaptable production systems, integrated with process analytical technologies (PAT) is critical to enhance scalability and ensure batch-to-batch consistency 165. Microfluidic mixing represents a production platform closely aligned with GMP requirements. The application of microfluidic bioprocessing enables precise control over particle size and uniformity in GMP settings, making it highly promising for clinical and commercial translation 166. Overall, significant challenges still remain in balancing obtaining GMP certification, controlling production costs, and improving production efficiency.

Safety is one of the important indicators for evaluating vaccines. Though biomimetic cell membrane-coated nanovaccines are high in biocompatibility and capability of targeting, there still a risk of off-target effects. Hence, enhancing the precision of vaccine targeting remains a critical priority and there is an urgent need for more innovative methods and strategies 167. Another safety concern regarding tumor vaccines is the long-term toxicity. Current research mainly focuses on short-term efficacy, while there is a serious lack of long-term toxicity data such as organ accumulation of toxic substances and immune tolerance. Additionally, the potential risk of immune-related adverse events (irAEs) represents a significant dimension of safety, with cytokine release syndrome (CRS) being the most frequently encountered condition in clinical practice. CRS often occurs in checkpoint inhibition or CAR-T therapy. However, nanovaccine could also trigger this syndrome 168. The excessive activation induced by potent adjuvants and highly immunogenic new antigens are likely to lead to the occurrence of CRS. Therefore, it is essential for developers and regulatory agencies to collaborate in establishing a comprehensive and robust regulatory framework 169.

Beyond safety concerns, the current regulatory system is limited by the absence of appropriate classification frameworks and a lack of standardized characterization methods 170. Some guidelines focus primarily on nanomaterial size as the key property. However, a more holistic view should include the characteristics of the particles as well as the chemical substances used during the preparation. Moreover, the dual nature of nanoparticles, which exhibit characteristics of both pharmaceutical drugs and medical devices, presents unique regulatory challenges, leading to underdeveloped and ambiguous regulatory pathways 171. Another challenge is the standardized characterization methods. To address the diversity of nanomaterials and their properties, unified standards must be established for evaluating their physical characteristics and stability 172. Thus, regulatory frameworks to oversee the development and approval of nanovaccines urgently need to be established by official and global regulatory bodies.

## Conclusions

This review summarizes the latest advancements in biomimetic nanovaccines for tumor immunotherapy, future prospects, and challenges in clinical application. Distinguished with traditional tumor vaccine, membranes of nanoparticle are extracted from various cells, such as tumor cells, DCs, erythrocytes and even hybrid cells. Due to the signal molecules and specific proteins on the membrane, it could be used to protect antigen from clearance and improve the efficiency of vaccines. Besides, nanovaccine combine with other therapy like chemotherapy or immune check point therapy could reduce side effects and improve therapeutic effect. Using sonodynamic and photothermal methods make nanovaccine release inner drugs more controllable. However, biomimetic cell membrane-coated nanovaccines are still in its infancy. Owing to these distinctive advantages, biomimetic nanovaccines represent a promising strategy to overcome current limitations in tumor immunotherapy, potentially emerging as a key for tumor therapy and prevention. Further research is warranted in the areas of industrial manufacturing, safety oversight, functional modifications, and fusion techniques.

## Figures and Tables

**Table 1:** Current approved or pipeline cancer vaccines

Vaccine	Type	Clinical stage	Tumor	Refs
Sipuleucel-T	Dendritic cells (DCs) vaccine	Approval	Prostate cancer	5
DOC1021	DCs vaccine	Approval	Glioblastoma	6
ELI-002 2P	Peptide vaccine	Phase 1 trial	Colorectal cancer (CRC), pancreatic ductal adenocarcinoma (PDAC)	1, 7
VGX-3100	DNA vaccine	Phase 2 trial	Human papillomavirus (HPV)	8
GX-188E	DNA vaccine	Phase 2 trail	HPV	9
mRNA-4157 (V940)	mRNA vaccine	Phase 2 trail	Melanoma	10
Autogene cevumeran	mRNA vaccine	Phase 1 trial	Pancreatic ductal adenocarcinoma (PDAC)	11

**Table 2:** The different types of cell membrane-coated biomimetic nanovaccines

Membrane	Antigen/Drugs	Adjuvant	Core	Combined therapy	Tumor models	Refs
Tumor cell membrane	TAAs	2'3'-cGAMP	PLGA	Radiotherapy	Melanoma, breast tumor	65
Tumor cell membrane	TAAs	Anti-CD40 single chain variable fragment	PLGA	-	Pancreatic tumor	66
DCs membrane	Neoantigen	-	Mesoporous silica framework	Phototherapy	Hepatocellular carcinoma	21
Bone marrow-derived dendritic cells (BMDCs) mixed with TEXs	Neoantigen, TAAs	-	Self-assembled ferrous ion NPs	-	Orthotopic glioma	67
Erythrocyte membrane	TAAs	Mannose	PLGA	-	Melanoma	68
Erythrocyte membrane,	iPSC	Mannose	Liposomes	-	Melanoma	69
Stem cell membrane	TAAs	CpG	PLGA	-	Melanoma	70
Hematopoietic stem and progenitor cell (HSPC) membrane	Cytarabine	-	Liposomes	Chemotherapy	Leukemia	71
Proinflammatory leukocytes membrane	Doxorubicin (DOX), LPCAT1 small interfering RNA	-	EYLN	Chemotherapy, gene therapy	Esophageal tumor	72
Bacterial outer membrane vesicles	BFGF	LPS	-	-	Melanoma	73
DCs membranes fused with bacterial E. coli cytoplasmic membranes and tumor cell membranes	TAAs, TSA	Bacterial membrane	F127 micelles	-	Melanoma	74
Macrophage membrane	-	-	shRNA-PEI-iRGD	Chemotherapy	Melanoma	75

**Abbreviations:** PLGA, poly (lactic-co-glycolic acid); TAA, tumor-associated antigen; DCs, dendritic cells; BMDCs, bone marrow-derived dendritic cells; TEX, tumor-derived exosome; NPs, nanoparticles; iPSC, induced pluripotent stem cell; HSPC, hematopoietic stem and progenitor cell; BFGF, basic fibroblast growth factor; LPS, lipopolysaccharide.

**Table 3:** Advantages and limitation of different types of membrane coated nanovaccines.

Membrane types	Advantages	Limitations
Tumor cell membrane	Cultured *in vitro* in large quantities, homologous targeting 76, antigen library 77	The effects of tumor microenvironment 78
DC membrane	Targeting lymph node 79	Short lives, insufficient delivery 80
Erythrocyte membrane	Escape immune clearance 81	Blood type 82, lack specific targeting 83
Macrophage cell membrane	Targeting lysis 84, accessible for tumors lacking vessels 85 and brain blood barrier (BBB)^86^	Slow traffic under intravenous injection 85
Stem cell membrane	Traverse biological barriers 87, express oncofetal antigens 88	Accumulation in liver 50
Bacterial outer membrane vesicles	Pathogen-associated molecular trigger high immune response 89	Maturation-induced uptake obstruction (MUO) 90
Hybrid membrane	Flexibility 74	Manufacturing

**Table 4:** Comparison of combined therapy in biomimetic nanovaccine.

Combined therapy	Advantages	Limitations
Chemotherapy	Eliminate systemic toxicity, reshape immunosuppressed TME, induce ICD releasing antigens 126	Limited by TME 127, free small molecule drugs lacking targeting ability 126
Immune checkpoint therapy	Reduce antibody dose, improve immunogenicity 128, disrupted co-inhibitory signal on APCs	Limited by TME 129
Photothermal therapy	Induced ICD releasing antigens 130, invasive	Reactive oxygen species (ROS) effects 131
Sonodynamic therapy	Penetrating tissue ability, induce ICD releasing antigens 132	ROS effects 133, radiation-induced damage
Gene therapy	Delivering drugs, enhancing CAR-T in solid tumor 134	Neurotoxicity 135, limited by TME
